# Strategies to Reduce Rebound Pain and Facilitate Early Recovery After Transforaminal Endoscopic Lumbar Discectomy

**DOI:** 10.3390/jcm14103529

**Published:** 2025-05-18

**Authors:** Yong Ahn

**Affiliations:** Department of Neurosurgery, Kyung Hee University Hospital at Gangdong, Kyung Hee University College of Medicine, Seoul 05278, Republic of Korea; ns-ay@hanmail.net; Tel.: +82-2-440-6147

**Keywords:** discectomy, endoscopy, lumbar vertebrae, minimally invasive surgical procedures, postoperative pain, recurrence

## Abstract

**Background**: Transforaminal endoscopic lumbar discectomy (TELD) is a minimally invasive and popular surgical method for the treatment of lumbar disc herniation. Although TELD offers favourable outcomes and enables fast recovery, some patients experience rebound pain and transient postoperative pain, which can delay rehabilitation and decrease patient satisfaction. **Methods**: This narrative review was conducted based on a comprehensive literature search of the MEDLINE database, supplemented by the author’s clinical experience. Relevant articles were identified using the keywords “rebound pain” and “transforaminal endoscopic lumbar discectomy” or “percutaneous endoscopic lumbar discectomy”. A thorough examination of rebound pain after TELD was performed by reviewing what has currently been published about its clinical traits. It was also compared with what could be observed in open lumbar discectomy and proposed preventive measures. **Results**: Rebound pain typically occurs within 2 weeks postoperatively and resolves spontaneously within 3 weeks. The proposed pathologies include inflammatory edema, transient ischemia, neural hypersensitivity, and increased pressure inside the disc. Risk factors include early unreasonable activity, incomplete release, and psychological predispositions. Rebound pain must be distinguished from recurrent herniation. Prevention strategies include adequate decompression, minimal neural irritation, postoperative medications, and early mobilization protocols. **Conclusions**: Rebound pain after TELD is self-limiting but has a clinical effect that may delay timely rehabilitation and raise concerns for surgeons and patients. Awareness and early recognition can enhance postoperative care and optimize clinical outcomes after TELD.

## 1. Introduction

As human life expectancy increases and greater emphasis is placed on quality of life, minimally invasive spine surgery (MISS) has gained increasing importance [1,2]. With patients seeking not only longer lives but also improved function and reduced surgical trauma, MISS techniques have gained widespread popularity and recognition across medical fields.

Full-endoscopic spine surgery is the ultimate realization of MISS [3,4,5,6,7]. Since Kambin [8] and Hijikata [9] introduced the posterolateral percutaneous lumbar disc decompression techniques in the mid-1970s, endoscopic spine surgery has undergone significant advancements. Transforaminal endoscopic lumbar discectomy (TELD) has become a representative endoscopic spine technique for treating lumbar disc herniation approached posterolaterally through the foraminal window and avoids injury to normal posterior musculoskeletal structures. Selective discectomy can be performed with minimal dural sac retraction under direct endoscopic visualization. The effectiveness of TELD compared with that of standard open lumbar discectomy has been scientifically validated through randomized controlled trials [10,11,12,13,14,15,16] and meta-analyses [17,18,19,20,21,22,23,24,25]. Because it offers less operative trauma, faster rehabilitation, and lower complication rates, TELD is increasingly being adopted for treating lumbar disc herniation [26,27,28,29].

Although MISS or endoscopic spine surgery is generally associated with reduced postoperative pain compared to open surgery [26,27,28,29,30], a subset of patients undergoing TELD may experience a unique and transient type of postoperative pain, often referred to as rebound pain. Considerable radicular pain, with or without back pain, can relapse a few days after symptom alleviation. It may last for several weeks and is relieved spontaneously. This transient discomfort usually does not affect the long-term clinical outcomes. However, patients and surgeons may worry about recurrence or incomplete procedures because of unexpected pain, leading to suspicion about the efficiency of the treatment. Therefore, this rebound pain phenomenon must be adequately elucidated when managing patients to help them return to their ordinary lives. Strategies to reduce rebound pain should be developed to facilitate postoperative management and rehabilitation. Although some studies have reported on rebound pain after TELD [31,32,33], no comprehensive reviews or management protocols have specifically addressed its pathophysiology or prevention.

This review aimed to provide a comprehensive overview of the characteristics and underlying mechanisms of rebound pain following TELD. Furthermore, it seeks to propose preventive measures and rehabilitation strategies to enhance postoperative patient care and improve overall treatment outcomes.

Although reports on rebound pain after TELD remain limited, this narrative review was conducted based on a comprehensive search of the MEDLINE database, supplemented by the author’s clinical experience. The following keywords were searched: “rebound pain” and “transforaminal endoscopic lumbar discectomy” or “percutaneous endoscopic lumbar discectomy”.

## 2. Definition of Rebound Pain After TELD

Rebound pain is a transient exacerbation of postoperative pain following the initial period of relief after TELD. It is different from recurrent disc herniation, which results from structural failure owing to its self-limiting nature and the absence of progressive neurological deficits [31,32,33].

**Figure 1:**
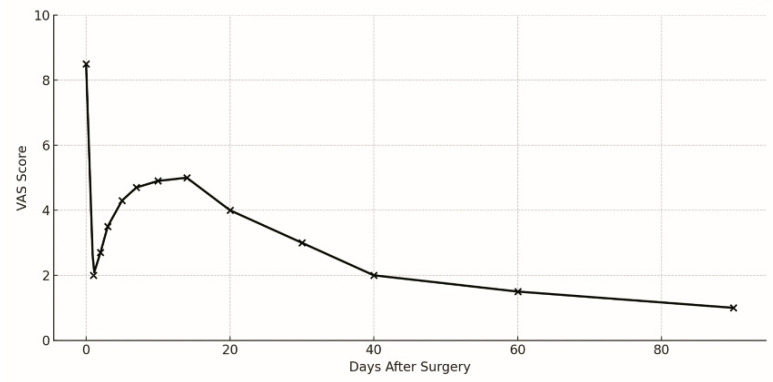
The clinical characteristics of rebound pain following transforaminal endoscopic lumbar discectomy (TELD). Rebound pain is defined as a transient recurrence of leg pain, with or without back pain, following initial postoperative improvement after TELD. Typically occurring within 2 weeks after surgery, the pain is milder than the preoperative level and resolves spontaneously within 3 weeks.

Clinically, rebound pain occurs within 2 weeks after surgery and gradually resolves within 3 weeks (Figure 1) [33]. Leg pain, with or without back pain, is the dominant symptom, milder than preoperative pain, often having a visual analogue scale (VAS) score of <6. However, rarely, patients might experience severe pain with a VAS score > 6, requiring closer clinical monitoring and symptomatic management [32,33]. In contrast to recurrent herniation or residual fragments, which might necessitate reoperation, rebound pain has a good response to conservative treatment with nonsteroidal anti-inflammatory drugs and physical therapy [33,34].

## 3. Pathogenesis of Rebound Pain After TELD

**Figure 2:**
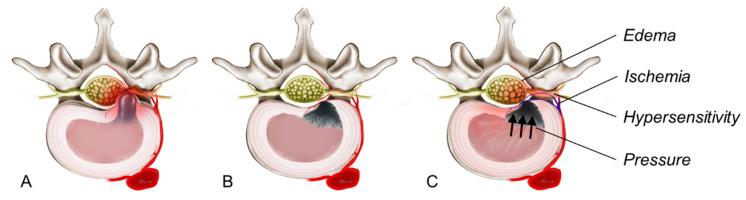
The proposed pathogenesis of rebound pain after transforaminal endoscopic lumbar discectomy (TELD). (**A**) A herniated lumbar disc causing severe radiculopathy by compressing the dural sac and nerve root. (**B**) Immediate postoperative status after a successful TELD. The herniated disc was selectively removed, and neural decompression was achieved. (**C**) The development of rebound pain is multifactorial. Despite successful decompression, inflammatory edema around the nerve root, ischemia–reperfusion injury, neural hypersensitivity, and increased intradiscal pressure contribute to transient symptom exacerbation.

Despite the unclear mechanism of rebound pain, several hypotheses have been proposed to explain its occurrence (Figure 2). Inflammatory changes following neural decompression, postoperative edema, and transient ischemia of the nerve root are implicated [32,33]. In addition, factors such as insufficient resting and premature mobility after surgery may exacerbate this condition. Zhang et al. reported that rebound pain occurred in 10.4% of patients undergoing percutaneous endoscopic lumbar discectomy. An early return to work (<45 days postoperatively) was suggested as a contributing factor [32]. Similarly, Lin et al. observed that rebound pain is self-limiting, usually resolving within 3 weeks and not significantly affecting long-term surgical outcomes [33].

### 3.1. Inflammatory Edema of the Nerve Root

Nerve root irritation during TELD, pre-existing inflammatory changes, and minor intraoperative trauma may contribute to postoperative inflammatory edema, leading to transient neural compression [32]. Small blood clots formed at the surgical site may further exacerbate inflammation and prolong the pain perception during recovery. In particular, a minor injury to the facet joint, most commonly during early postoperative activity, has been implicated in rebound pain. Inflammatory mediators, such as interleukin (IL)-6 and tumour necrosis factor (TNF)-α, released following decompression may lead to hypersensitivity of the nerve root, worsening postoperative pain. Histological findings show that edema at the surgical decompression site, which can transiently compress neural structures, may be responsible for rebound pain [34].

### 3.2. Vascular and Microcirculatory Changes

In addition to inflammatory changes, other contributing factors include alterations in the nerve root microcirculation. Decompression can disrupt the local vascular supply to the nerve root, resulting in transient ischemia–reperfusion injury [32]. This is commonly seen in other neural decompression types and may explain the delayed rebound pain effect. Ischemia–reperfusion injury leads to oxidative stress and endothelial dysfunction, which can increase pain sensitivity and extend recovery [33].

### 3.3. Neural Hypersensitivity Due to the Disruption of Pain Pathways

Another possible mechanism involves neural hypersensitivity due to the sudden nerve root decompression. Rapid pressure reduction may lead to temporary hypersensitivity of the dorsal root ganglia, altering normal pain transmission pathways and causing exaggerated pain responses despite the absence of structural compression [33]. This phenomenon is consistent with findings in other neuropathic pain conditions, where abrupt changes in the neural load can lead to temporary hyperexcitability of pain-sensing neurons [34].

### 3.4. High Intradiscal and Epidural Pressure After Discectomy

Although not widely discussed in the literature, high intradiscal and epidural pressures following successful discectomy may also contribute to the development of rebound pain. During the early postoperative period, before annular healing is complete—a process believed to be slow and variable [35,36]—spinal movements may increase pressure within the discectomy cavity, potentially exacerbating transient pain. This hypothesis is backed by evidence that rebound pain occurs mainly following disc removal procedures. When more disc material is removed in a normal discectomy and a posterior laminectomy is performed, the resulting larger space is thought to further buffer pressures, thereby reducing the risk of rebound pain. This phenomenon occurs because TELD preserves more of the central nucleus and results in a relatively higher pressure in the intervertebral space (compared with conventional discectomy), which may make it more susceptible to rebound pain.

These findings highlight the complexity of rebound pain, which is influenced by inflammatory, vascular, neural, and biomechanical factors. Further research is needed to elucidate the underlying mechanisms, which will aid in the effective management of affected patients. Understanding how to modify the surgical procedure perioperatively is also essential.

## 4. Incidence of and Risk Factors for Rebound Pain After TELD

Rebound pain is a well-recognized but often under-reported phenomenon following TELD. Although the percentage of patients experiencing this condition varies between studies, it is commonly observed early in the treatment course. Zhang et al. [32] found that 10.4% of patients experienced rebound pain after percutaneous endoscopic lumbar discectomy. In another study, Lin et al. [33] indicated that approximately 5.8% of patients reported rebound pain after full-endoscopic lumbar discectomy. Although most cases were self-limiting and resolved within 1 month, some patients required prolonged conservative management. These findings suggest that rebound pain is not an uncommon postoperative condition and could be a source of concern for both patients and surgeons.

Several risk factors are proposed to cause rebound pain. In particular, early postoperative activity has been identified as an important risk factor. Premature return to work, particularly within 45 days postoperatively, may increase the risk of rebound pain [32]. A possible explanation is that this activity subjected the healing surgical site to increased mechanical stress, leading to a temporary irritation of the nerve root and soft tissues. Likewise, inadequate postoperative rest is associated with a high risk of rebound pain, highlighting the importance of educating patients about early activity modification.

However, some studies have suggested that an early return to activity does not necessarily compromise surgical outcomes. Carragee et al. [37] reported that lifting postoperative activity restrictions after lumbar discectomy did not increase the incidence of complications in a prospective cohort of 50 patients. Similarly, Bono et al. [38], in a randomized controlled trial, found that returning to work after 2 weeks was not associated with worse outcomes or a higher risk of reherniation compared to 6 weeks of restricted activity. These findings indicate that although careful adjustment of activity may help reduce transient postoperative pain, rigid or prolonged restrictions may not always be necessary.

Rebound pain may also be due to surgical factors, such as the amount of disc material excised, residual fragments, and minor trauma to the facet joint. In situations where only a small amount of the disc is removed, the disc will still be under pressure, particularly while moving, and pain rebounds after surgery. This could clarify the prevalence of rebound pain in patients undergoing TELD, where the preservation of the central nucleus may lead to increased pressure in the discectomy cavity. In contrast, conventional open discectomy, which involves greater removal of the disc material and typically involves posterior laminectomy, may be less susceptible to increased pressure, thus accounting for the relatively low incidence of rebound pain.

Certain patients may experience rebound pain due to the response of vascular and inflammatory biological factors. The hypersensitivity of the nerve root, which is due to the increased release of inflammatory mediators such as IL-6 and TNF-α, can promote pain. Furthermore, after surgery, ischemia–reperfusion injury can make the nerves overly sensitive, which can worsen pain in some patients. These mechanisms indicate that individual variations in inflammatory and vascular responses to surgery may influence the risk of rebound pain.

Patient-related factors, including preoperative pain levels, baseline inflammatory status, and psychological factors, such as anxiety and pain catastrophizing, may also affect the rebound pain. Those patients who experience more severe preoperative pain are likely to experience negative transient pain postoperatively. In addition, patients who have been diagnosed with chronic pain conditions or have experienced pain sensitivity may experience rebound pain more than those without these conditions, even in the absence of structural damage. Considering all of the above, the incidence and risk factors of rebound pain after TELD are influenced by surgical, biological, and patient factors. Although rebound pain is typically self-limiting with no lasting consequences, being aware of the factors is essential to enhance postoperative care.

Ensuring adequate patient education, encouraging a gradual return to activity, and finding ways to limit inflammation and vascularization could reduce the risk of rebound pain and improve satisfaction and outcomes of TELD.

## 5. Differential Diagnosis of Rebound Pain

Rebound pain is usually distinguished from recurrent disc herniation and other postoperative complications following TELD. Postoperatively, these conditions may coexist because of overlapping symptoms. Rebound pain and recurrent disc herniation may cause leg pain, with or without back pain, which can create confusion. Their mechanisms of action and development differ, making their diagnosis and management complex.

Rebound pain is a temporary condition that usually arises within the first 2 weeks following surgery and improves within 3 weeks. In most cases, it is characterized by mild to moderate pain that does not exceed preoperative pain levels; however, some cases have been reported to have even higher VAS scores. Rebound pain does not result in new nerve problems such as recurrent disc herniation. It can be alleviated by physical therapy and use of over-the-counter painkillers. Rebound pain usually improves gradually, and symptoms do not worsen over time.

In recurrent disc herniation, the new or residual disc material undergoes further extrusion, causing pain that worsens and lasts longer than that associated with the surgery. The incidence of recurrent disc herniation after TELD ranges from 0.5% to 12.5% [39,40,41,42,43,44]. Pain from recurrent herniation usually lasts longer than a month and may worsen, particularly with activity. Unlike rebound pain, recurrent disc herniation can occur at any time postoperatively and may present with radicular symptoms that mirror the preoperative complaints [43,44]. In some cases, patients may report a sudden recurrence of pain following the initial period of postoperative relief. If pain does not follow the expected resolution pattern of rebound pain or worsens progressively over time, further evaluation with magnetic resonance imaging (MRI) is warranted to assess recurrent disc pathology.

Differentiating between these conditions crucially depends on the clinical observations and response to treatment. If the symptoms begin to improve during the early treatment period, rebound pain is likely. If the pain does not resolve after 4 weeks, worsens, and causes symptoms related to the radicular nerves, a recurrent herniation might be suspected. In such instances, MRI may be considered to assess new disc extrusion or compression phenomena [45].

Patients with postoperative pain should also be evaluated for residual disc fragments, epidural hematomas, and infections. Residual disc fragments may lead to persistent symptoms similar to recurrent herniation; however, they typically manifest immediately after surgery without a pain-free interval [45,46]. Although relatively rare, postoperative hematoma may present with progressive neurological deterioration and require urgent imaging and intervention if suspected [47]. Postoperatively, infections such as discitis should be considered in patients experiencing severe pain and fever and have high levels of inflammatory markers such as erythrocyte sedimentation rate and C-reactive protein [48].

Because postoperative pain can be confused with other pain types, a systematic assessment approach is essential. Typical rebound pain can be managed conservatively through reassurance and symptomatic treatment. Those exhibiting persistent, worsening, or atypical symptoms require further investigations. Rebound pain can be distinguished from other serious postoperative complications by careful monitoring of the clinical course and selective imaging.

## 6. TELD Versus Open Lumbar Discectomy: Differences in Rebound Pain

**Figure 3:**
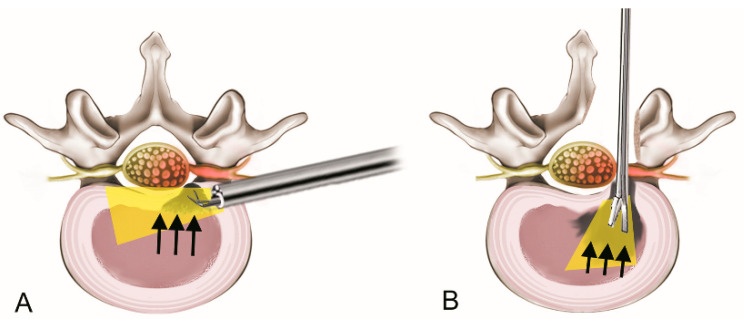
A comparison of surgical principles between transforaminal endoscopic lumbar discectomy (TELD) and open lumbar discectomy. (**A**) TELD involves limited disc removal without laminectomy and preserves the central nucleus, which may retain the intradiscal pressure (arrows). This may lead to transient neural irritation during the recovery period. (**B**) In contrast, open discectomy typically involves more radical disc removal with posterior decompression (e.g., laminectomy), which creates a greater space for postoperative edema and reduced residual pressure (arrows).

Rebound pain following TELD differs from that observed following conventional open lumbar discectomy due to fundamental differences in the surgical technique, extent of disc removal, and structural changes following each procedure. Although both procedures aim to relieve nerve root compression, the minimally invasive nature of TELD affects unique biomechanical and physiological factors that may predispose patients to rebound pain more frequently than open discectomy (Figure 3).

A key difference between the two procedures is the extent of disc removal. The main focus of TELD is the selective removal of the extruded or sequestered disc fragment while preserving most of the nucleus pulposus and annulus. Compared with TELD, open lumbar discectomy generally involves more extensive removal of the disc, including the partial evacuation of the nucleus pulposus. In TELD, limited removal of the disc may maintain its height and function; however, this may contribute to residual intradiscal pressure. Persistent pressure can irritate the dural sac and nerve root during rehabilitation, resulting in significant pain and discomfort.

In addition, the risk of reherniation may increase following TELD because the preserved disc material retains its potential to migrate to the foramen or canal following postoperative movement.

Another factor to consider is the potential presence of hidden internal disc fragments. Owing to the working channel approach used in TELD, access to deeper areas of the disc space is inherently more limited than that in open surgery. This limitation increases the likelihood that small fragments of the herniated nucleus pulposus remain undetected within the annulus. When fragments exert intermittent pressure on the nerve root or an inflammatory effect, it may induce pain that appears to rebound postoperatively. Conversely, open discectomy allows for the direct evacuation of the entire disc space, decreasing the likelihood of retained fragments and reducing postoperative discomfort.

The effect on the posterior spinal parts was also quite different between the two procedures. Open lumbar discectomy often involves complete or partial laminectomy, which decompresses the spinal canal posteriorly and provides additional space to the nerve root. This increased widening may act as a buffer against postoperative changes in pressure at the epidural and intradiscal sites, which could result in rebound pain. However, the laminar bone and ligamentum flavum remain intact in TELD because they do not involve posterior element resection. Although this preservation helps keep the spine stable, the compensatory space may be insufficient in the spinal canal for postoperative edema or temporary augmentation of epidural pressure. This may have caused an increase in the rebound pain.

These differences underscore the unique mechanisms by which rebound pain may arise following TELD compared with open lumbar discectomy. Despite the advantages of TELD, such as reduced tissue trauma, faster recovery, and preservation of spinal stability, it has some disadvantages that may expose patients to transient postoperative pain. Knowledge of these differences guides the selection of the right patient for surgery and provides guidance on postoperative rehabilitation that minimizes rebound effects, but to obtain the benefits of minimally invasive surgery. More studies comparing the incidence and severity of rebound pain between TELD and open discectomy are required to refine postoperative management approaches and improve patient outcomes.

## 7. Effect of Rebound Pain on Rehabilitation and Patient Satisfaction

Several studies have addressed the clinical effect of postoperative pain following lumbar discectomy. In a prospective cohort study, Ostelo et al. [49] demonstrated that patients with higher levels of early postoperative pain had delayed return to function and lower satisfaction scores 6 months postoperatively. Similarly, Ziegler et al. [50] indicated early postoperative pain intensity as a strong predictor of long-term disability and suboptimal patient-reported outcomes.

Postoperative pain can result in hesitation or avoidance of physical activity, reduced compliance with rehabilitation programmes, and increased fear-avoidance behaviour, which may contribute to prolonged recovery time [41,42]. Furthermore, the use of opioids in the early postoperative period is often required to manage severe rebound pain, which may hinder functional recovery and increase the risk of chronic pain [32,33].

Rebound pain may arise following the resolution of local anesthesia or nerve block postoperatively, which can significantly affect rehabilitation and patient satisfaction [32,33]. This phenomenon is particularly concerning in MISS, where effective pain management is crucial for optimal recovery.

Rebound pain can hinder early mobilization and active engagement in rehabilitation therapy. High postoperative pain levels can cause less movement and decreased muscle use, which can slow functional recovery. In addition, heightened pain perception may decrease a patient’s desire to participate in rehabilitation exercises, leading to reduced compliance with the recovery protocol [32,33]. Consequently, chronic disability and late return to daily activities, with poor long-term outcomes, may occur.

As regards patient satisfaction, rebound pain can lead to increased frustration and anxiety. Unexpected postoperative pain spikes can induce a sense of dissatisfaction even if the surgery is successful. Patients who experience severe rebound pain may perceive their overall surgical experience as unfavourable, potentially diminishing their trust in the healthcare provider. Moreover, inadequate pain control can increase patients’ reliance on opioid analgesics, raising concerns regarding potential side effects and dependency [32,33].

To prevent rebound pain from disrupting rehabilitation and causing low patient satisfaction, the effects of diminishing pain relief were reconsidered [32,33]. Teaching patients what to expect regarding postoperative pain and the use of multimodal analgesia can help set realistic expectations. When rebound pain is addressed, patients are likely to participate in rehabilitation sooner—which enhances their recovery—and express greater satisfaction with their surgery.

## 8. Strategies to Prevent Rebound Pain After TELD

Preventing rebound pain following TELD requires a comprehensive, multimodal approach that incorporates preoperative, intraoperative, and postoperative strategies. Meticulous surgical technique, combined with optimized perioperative management, is essential to minimize nerve irritation and inflammatory responses that contribute to rebound pain.

### 8.1. Preoperative Strategies

Effective prevention of rebound pain begins before the surgery itself. First, a thorough preoperative review of imaging studies, including MRI and CT, is critical. Careful evaluation helps in planning an approach route that minimizes tissue irritation related to access and endoscopy. Selecting the optimal surgical trajectory based on anatomical considerations reduces the risk of intraoperative neural injury or mechanical irritation.

Second, preoperative patient education is essential. Patients should be informed about the possibility of temporary rebound pain or postoperative discomfort, even after successful decompression. Setting realistic expectations and teaching coping strategies can help minimize anxiety and improve postoperative compliance and satisfaction.

Third, optimizing the preoperative inflammatory status is also important. Patients with ongoing systemic inflammation or uncontrolled diabetes, for example, may require additional medical management before surgery to reduce postoperative inflammatory responses that could exacerbate rebound pain.

### 8.2. Intraoperative Strategies

One of the most critical aspects of rebound pain prevention is ensuring complete decompression of the pathological structures rather than merely addressing the most prominent or exposed lesions. The concept of “removing the entire iceberg” rather than just its visible tip is fundamental in endoscopic spine surgery [34]. If only the part of the epidurally protruding disc seen during surgery is targeted, the residual hidden disc tissue may cause persistent or recurrent pain. Therefore, surgeons should check for any unidentified debris that may cause inflammation and neural irritation using endoscopic disc probing or pressurized disc irrigation before the surgery ends.

The second basic principle is to achieve a sufficient decompression margin. This means not only removing disc fragments but also ensuring adequate resection of the bone at the lateral recess and foramen where necessary. Appropriately enlarging the bony space prevents nerve root compression due to postoperative neural edema. This is especially true in cases of foraminal window narrowing. Dynamic irritation can cause both irritation and recurrence. Ensuring precise and sufficient bony margin resection ensures lasting decompression and does not cause irritation or rebound pain [51].

In addition to bony decompression, effective neural release from soft tissue adhesion is also important [5]. Simple exposure of the nerve root alone is insufficient. Thus, the nerve should be freed entirely to move without restrictions. Annular anchorage at the herniation point must be released to facilitate the complete removal of herniated disc fragments that might be tethered to the nerve. Without this step, residual mechanical irritation and traction forces can continuously induce postoperative pain, even after the visible compression has been relieved.

Minimizing the device manipulation of the neural structure must also be considered. Pain related to surgical access can be minimized using an appropriate transforaminal approach. A pre-emptive epidural and nerve root block can help decrease surgery-related inflammation. Moreover, using clear and stable endoscopic vision with optimal orientation prevents unnecessary damage to the neural structure. Whenever possible, the surgeon should avoid direct contact with the nerve root or dural sac. Only the pathological disc material and inflamed soft tissue should be selectively excised. By being selective, normal structures that are otherwise nonpathological are preserved.

### 8.3. Postoperative Strategies

Adequate postoperative pain control is vital in reducing rebound pain. The use of multimodal pharmacological methods targeting different pain mechanisms can help prevent postoperative pain. Gabapentinoids, such as pregabalin and gabapentin, stabilize the electrical activity of nerves for the treatment of neuropathic pain. Corticosteroids, particularly methylprednisolone, can reduce nerve root inflammation and postoperative radicular symptoms. Nonsteroidal anti-inflammatory drugs such as celecoxib can help inhibit the release of inflammatory cytokines.

Nerve root blocks or epidural injections performed at the end or immediately after surgery using local anesthetics and steroids may reduce the level of inflammation and flare-up of pain [52]. This helps avoid an early postoperative inflammatory response, which is mainly responsible for rebound pain.

Beyond pharmacological strategies, postoperative care should focus on early mobilization to prevent the progression of nerve root edema. Encouraging patients to mobilize early after surgery facilitates venous and lymphatic drainage and reduces perineural edema. Gradual resumption of activity is important to avoid sudden weight-bearing stress that can irritate the nerve. Patients should be educated on activity modification to prevent excessive mechanical strain at the surgical site.

Physical therapy promotes nerve root adaptation and prevents secondary musculoskeletal complications, such as muscle spasms. Guided exercises, which help stabilize the core and control lumbar movements, can alter spinal biomechanics, thereby preventing tissue injury or recurrent pain. A rehabilitation programme tailored to individual patients may help patients recover and make them functional [53].

By integrating these intraoperative and postoperative strategies, post-TELD rebound pain can be effectively minimized, leading to improved patient satisfaction and long-term surgical success.

## 9. Conclusions

Post-TELD rebound pain is a transient yet clinically significant phenomenon that can affect postoperative recovery, rehabilitation progress, and overall patient satisfaction. Although it arises from temporary nerve root inflammation and vascular changes rather than surgical failure, its effects can hinder early mobilization and reduce treatment adherence. To minimize the effect of rebound pain, effective prevention and management strategies— such as comprehensive intraoperative decompression, meticulous nerve release, multimodal analgesia, and structured postoperative rehabilitation—must be employed. Prompt management of rebound pain helps in functional recovery and promotes patient trust in endoscopic spine surgery. Future prospective studies are needed to help standardize protocols to improve patient outcomes.

## Data Availability

No new data were created or analyzed in this study. Data sharing is not applicable to this article.

## Abbreviations

The following abbreviations are used in this manuscript:

TELD	Transforaminal endoscopic lumbar discectomy
MISS	Minimally invasive spine surgery
VAS	Visual analogue scale
IL	Interleukin
TNF	Tumour necrosis factor
MRI	Magnetic resonance imaging
